# Effect of online hemodiafiltration on quality of life, fatigue and recovery time: a systematic review and meta-analysis

**DOI:** 10.1007/s10047-024-01459-7

**Published:** 2024-07-27

**Authors:** Maurizio Bossola, Ilaria Mariani, Manuela Antocicco, Gilda Pepe, Enrico Di Stasio

**Affiliations:** 1 Servizio Emodialisi, Dipartimento Di Scienze Mediche E Chirurgiche, Università Cattolica del Sacro Cuore, Rome, Italy; 2 Policlinico Universitario Fondazione Agostino Gemelli IRCCS, Rome, Italy; 3 Dipartimento Scienze Dell’invecchiamentoNeurologiche, Ortopediche E Della Testa-Collo, Universitario Fondazione Agostino Gemelli IRCCS, Rome, Italy; 4 Dipartimento Di Scienze Biotecnologiche Di Base, Cliniche Intensivologiche E Perioperatorie, Università Cattolica del Sacro Cuore, Rome, Italy

**Keywords:** Hemodiafiltration, Quality of life, Fatigue, Time of recovery after dialysis, Hemodialysis

## Abstract

The present systematic review and meta-analysis aims to compare the effect of bicarbonate hemodialysis and HDF on quality of life (QoL), fatigue, and time to recovery in end-stage renal disease (ESRD) patients. Searches were run on January 2024 and updated on 3 March 2024 in the following databases: Ovid MEDLINE (1985 to present); Ovid EMBASE (1985 to present); Cochrane Library (Wiley); PubMed (1985 to present). Ten articles were fully assessed for eligibility and included in the investigation. Compared to HD, online HDF had a pooled MD of the mental component score (MCS) of the SF-36 of 0.98 (95% CI − 0.92, 2.87; *P* = 0.31). and of the physical component score (PCS) of 0.08 (95% CI − 1.32, 1.48; *P* = 0.91). No significant heterogeneity was observed (Chi^2^ = 4.85; *I*^2^ = 38%; *P* = 0.18 and Chi^2^ = 3.85; *I*^2^ = 22%; *P* = 0.28, respectively). Studies that compared the QoL assessed through the Kidney Disease Questionnaire or KDQOL-SF and show that HDF does not improve QoL when compared with HD, in most studies. In five out of seven studies, HDF was not significantly more effective than HD in improving fatigue. The length of the recovery time resulted in similar in patients receiving HDF and HD in all studies included in the present review. HDF is not more effective than HD in improving QoL and fatigue and in reducing the length of time of recovery after dialysis.

**Supplementary Information:**

The online version contains supplementary material available at 10.1007/s10047-024-01459-7.

## Introduction

Quality of life (QoL) is an important concept in the field of medicine, and it is correlated to physical and psychological well-being. It can be affected by chronic conditions such as end-stage renal disease, especially if the patient is on chronic hemodialysis. It is poorer in these subjects than in the general population and in comparison to that of individuals with other chronic diseases [1–3]. In addition, low QoL is associated with an increased risk of mortality in such patients [4]. Therefore, therapeutic strategies aimed at improving the QoL of patients receiving maintenance hemodialysis are urgently needed.

One of the most common, distressing, and debilitating symptoms contributing to poor quality of life is fatigue [5–14]. Fatigue is a complex phenomenon that involves physical, psychological, and emotional components, described as feeling tired and needing rest or sleep after the dialytic session and the following day. Other common descriptors of hemodialysis-related fatigue include being worn out or drained or exhausted. Its incidence among dialysis recipients is high, ranging from about 50 to 85%. Fatigue starts at the initiation of the dialytic session, progressively increases during the dialytic treatment and peaks at the end and in the following hours [15]. Fatigue conditions the time of recovery after dialysis (TIRD), a measurement expressed in minutes that is significantly associated with QoL [16].

Online hemodiafiltration (HDF) is a dialysis modality that combines diffusive and convective clearance of uremic solutes and is associated with better removal of both small and middle molecules [17–19]. HDF improves intradialytic haemodynamic stability, reduces markers of inflammation and oxidative stress, and is associated with better control of blood pressure over time and a significant reduction of overall and cardiovascular-related mortality [17–19]. While it has been hypothesized that HDF use could improve patients’ QoL, this is not yet fully understood.

The present systematic review and meta-analysis aim to compare the effect of bicarbonate hemodialysis and HDF on QoL, fatigue, and time to recovery in end-stage renal disease (ESRD) patients.

## Methods

The present analysis was conducted in accordance with PRISMA (Preferred Reporting Items for Systematic Reviews and Meta-Analyses) guidelines and was pre-registered in the international prospective register of systematic reviews (PROSPERO; ID CRD42022373584).

### Eligibility criteria

Randomized, crossover or parallel studies were considered for inclusion if they were written in English and: (1) included adult patients (over 18 years of age) with ESRD on chronic hemodialysis/HDF since at least 6 months; (2) compared bicarbonate hemodialysis vs HDF; (3) reported on QoL through the assessment of the physical component summary score (PCS) and the mental component summary score (MCS) of the Short Form Health Survey with 36 questions (SF-36) or the Kidney Disease Quality of Life Short Form (KDQOL-SF) or the Kidney Disease Questionnaire or other tools; (4) reported on fatigue or post-dialysis fatigue; (5) reported time to recovery after dialysis (TIRD).

Studies on pediatric patients, pre-dialysis patients, acute kidney injury patients, ESRD patients with other renal replacement therapy modalities such as peritoneal dialysis and transplant were excluded.

### Information sources and search strategy

A medical librarian performed comprehensive searches to identify studies that compared the effect of HDF and hemodialysis on QoL or fatigue or TIRD. Searches were run on January 2024 and updated on 3 March 2024 in the following databases: Ovid MEDLINE (1985 to present); Ovid EMBASE (1985 to present); Cochrane Library (Wiley); PubMed (1985 to present). Search terms included “hemodialysis/haemodialysis” AND “hemodiafiltration” AND “online hemodiafiltration” AND (“quality of life” OR “QoL” OR “life quality” OR “HRQOL”) AND (“SF-36” OR “Short Form (36] Health Survey” OR “36-Item Short Form Survey” OR “short form (SF)-36 health status”) AND (“SF-12” or “Short Form (12) Health Survey” or “12-Item Short Form Survey” or “short form (SF)-12 health status”) AND “mental component score” OR “MCS” OR “physical component score” OR “PCS” AND “Kidney Disease Questionnaire” OR “KDQOL-SF” AND “fatigue” OR “post-dialysis fatigue” AND “time of recovery” OR “time of recovery after dialysis”.

### Selection process and data collection process

**Fig. 1:**
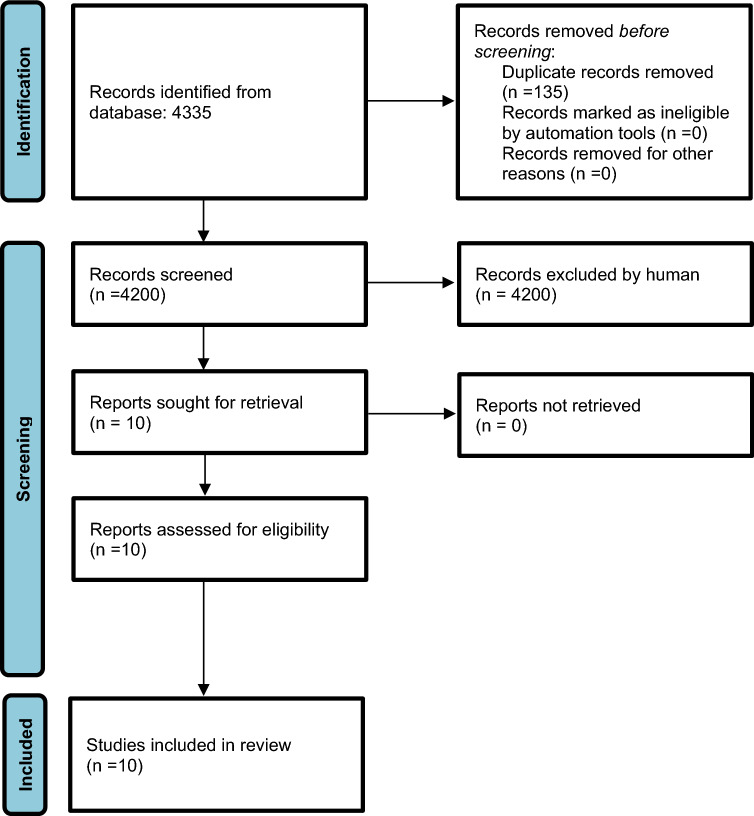
Preferred Reporting Items for Systematic Reviews and Meta-Analyses (PRISMA) flowchart of our analysis

Database searches screening and exclusion of duplicated results were performed by a qualified medical librarian. Two investigators screened the initial search results for inclusion and performed data extraction independently. Disagreements were resolved by a third author, who also checked the extracted data for accuracy. Full text for the selected studies was pulled for a second round of eligibility screening. Reference lists of articles were also searched to identify other relevant studies. The PRISMA flow diagram outlining the study selection process is available in Fig. 1.

### Data items

The primary outcome was mean scores and SD of the QoL and fatigue assessments and the length of time of recovery after hemodialysis.

### Quality assessment and risk of *bias*

The quality of reporting for each randomized, parallel study was performed by two researchers using the Quality Assessment Tool of Controlled Intervention Studies of the National Institutes of Health [20]. The quality of reporting of each randomized, cross-over study was performed by two researchers using the Revised Cochrane Risk of Bias (RoB) tool for cross-over trials as proposed by Ding et al. [21]. This tool explores 9 items: 1. appropriate cross-over design; 2. the randomized order of receiving treatment; 3. carry-over effects; 4. unbiased data; 5. allocation concealment; 6. blinding; 7. incomplete outcome data; 8. selective outcome reporting; 9. other biases. Based on the signaling questions provided in the RoB tool, each of the proposed domains received a rating that was either “low risk of bias”, “high risk of bias” or “some concerns of bias” or “unclear”. The authors followed the guidelines provided by the Cochrane community. Disagreements between authors were resolved through discussion. If no consensus could be reached, a third author (EDS) was consulted who reached a final verdict based on the comments of both authors and an independent screening of the articles that were the subject of discussion.

### Statistical analysis/synthesis methods

The primary outcome was the mean ± SD score of the tools used to assess QoL and fatigue, and the mean ± SD of the length of the TIRD. Statistical heterogeneity among studies was assessed with Cochran’s *Q* and quantified with Higgins *I*^2^ statistic [36–40]. Because of low heterogeneity (*I*^2^ = 0%), data were analyzed using a fixed effects (Mantel–Haenszel method) model approach [37]. Publication bias was assessed graphically using funnel plots. Statistical analysis was performed by using the Statistical Package for Social Science (SPSS 22.0; SPSS Inc, Chicago, IL, United States) and Microsoft Excel (Version 16.45).

### Certainty assessment

Overall evidence was qualified using Grading of Recommendations Assessment, Development and Evaluation (GRADE) working group guidelines for both cross-over and parallel-group observational studies. Results of the quality of evidence criteria are reported in Table 1S and 2S.

## Results

### Literature search

Four-thousand and three hundred thirty-five studies records were identified through database and hand searches. Of these, 135 were excluded based on duplicated data, and 4200 titles and abstracts were evaluated. Ten articles were fully assessed for eligibility and included in the investigation [22–31]. The flowchart of the study is shown in Fig. 1. We divided the analysis in three sections: (1) Comparison of HD and online HDF in terms of quality of life assessed through the PCS and the MCS of SF36 or SF12; (2) Comparison of HD and online HDF in terms of quality of life assessed through the Kidney Disease Questionnaire or KDQOL-SF; (3) Comparison of hemodialysis and hemodiafiltration in terms of fatigue and/or TIRD.

### Characteristics of the studies included

#### Comparison of HD and online HDF in terms of quality of life assessed through the PCS and the MCS of SF36 or SF12

Five studies were included [22–26]. Overall, 2757 patients were included. The number of patients in each individual study ranged from 24 to 1463. Three studies were randomized parallel controlled [23, 24, 26], two studies were randomized crossover [22, 25]. The length of studies ranged from 4 to 24 months and their description is presented in Table 1.

**Table 1:** Studies comparing the effect of HD and HDF on quality of life assessed through the physical component score (PCS) and the mental component score (MCS) of SF36 or SF12

	Type of study	Number of patients	Assessment of QoL	Duration	Main results (QoL)
Kantartzi et al., 2013^5^	Randomized, cross-over	24	PCS and MCS	12 months	No difference between HD and HDF
Mazairac et al., 2013^6^	Randomized, parallel	714	PCS and MCS	24 months	No difference between HD and HDF
Morena et al., 2017^7^	Randomized, parallel	261	PCS and MCS	24 months	No difference between HD and HDF except for MCS which tended to be higher in the HD group at 24 months (*P* = 0.06)
Smith et al., 2017^8^	Randomized, cross-over	100	PCS and MCS	4 months	No difference in PCS scores (33 ± 9 for both HDF and HD; *P* = 0.09) or MCS scores (44 ± 11 vs 43 ± 12 for HDF and HD, respectively; *P* = 0.6)
Pecoits-Filho et al., 2021^9^	Randomized, parallel	195	PCS and MCS	6 months	No difference between HD and HDF

#### Comparison of HD and online HDF in terms of quality of life assessed through the kidney disease questionnaire or KDQOL-SF

Four studies were included [27–30]. Overall, 1424 patients were included. The number of patients in each individual study ranged from 47 to 1013. Two studies were randomized parallel controlled [27, 29], two studies were randomized crossover [28, 30]. The length of studies ranged from 8 to 24 months and their description is presented in Table 2.

**Table 2:** Studies comparing the effect of HD and HDF on quality of life assessed through the Kidney Disease Questionnaire or Kidney Disease Quality of Life Short Form 36 (KDQOL-SF)

	Type of study	Number of patients	Assessment of QoL	Duration	Main results (QoL)
Ward et al., 2000	Randomized, parallel	45	Kidney disease questionnaire	12 months	No difference between HD and HDF
Schiffl et al., 2007	Randomized, cross-over	76	Kidney disease questionnaire	13 months	Partial improvement of QOL with HDF
Karkar et al., 2015	Randomized, parallel	72	KDQOL-SF	24 months	The HDF group achieved a higher satisfaction level than the HF group (*P* < 0.0001) with less cramps (3 ± 5 vs. 55 ± 8), itching (9 ± 10 vs. 48 ± 10), joint pain and stiffness (24 ± 10 vs. 83 ± 8), and improvement in general mood (94 ± 9 vs. 28 ± 16), sexual performance (57 ± 10 vs. 5 ± 5), and social activity (82 ± 9 vs. 15 ± 8)
Aichi et al., 2022	Randomized, cross-over	47	KDQOL-SF	8 months	The switch from bicarbonate hemodialysis to HDF did not lead to a significant improvement of quality of life

#### Comparison of HD and online HDF in terms of fatigue and recovery time after hemodialysis

Three studies compared hemodialysis and hemodiafiltration in terms of fatigue [22, 27, 29], and three studies in terms of TIRD [25, 26, 31]. In the three studies comparing hemodialysis and hemodiafiltration in terms of fatigue, the measurement used to assess fatigue was the SF-36 Vitality Scale in one study [22], the Kidney Disease Questionnaire in one study [27], the KDQOL questionnaire in one study [29], two studies were randomized, parallel and one study was randomized, cross-over. The length of randomized studies ranged from 12 to 24 months (Table 3).

**Table 3:** Studies comparing the effect of online hemodiafiltration (HDF) and bicarbonate hemodialysis (HD) on fatigue in end-stage renal disease patients

	Type of study	Number of patients	Intervention	Duration	Measurement	Main results
Ward et al., 2000	Randomized, parallel	45	Post-dilution HDF vs high-flux HD	12 months	Kidney disease questionnaire	Fatigue score from baseline to 12 months was 4.6 ± 0.3 to 4.9 ± 0.4 in HDF and 4.7 ± 0.3 to 4.9 ± 0.3 in HD; *P* = 0.083
Kantartzi et al., 2013	Randomized, cross-over	24	Post-dilution HDF vs low-flux HD	12 months	SF-36 Vitality scale	HD = 40 [25–53.7] and HDF 42.5 [30–60]*; *P* = 0.147
Karkar et al.,2015	Randomized, parallel	72	Post-dilution HDF vs high-flux HD	24 months	Modified KDQOL-SF (scale ranging from 0 to 100)	General fatigue 57 ± 12 with HD and 6 ± 6 with HDF (< 0.0001); Post-dialysis fatigue 61 ± 18 with HD and 10 ± 9 with HDF (< 0.0001)

*hHD* high-flux hemodialysis, *lHD* low-flux hemodialysis, *PD* peritoneal dialysis, *POMS* Profile of Mood States, *PDF* post-dialysis fatigue *Median [IQR]

In the three studies comparing hemodialysis and hemodiafiltration in terms of TIRD, the measurement used was a time of recovery assessed in minutes [25, 26, 31]. Two studies were randomized cross-over [25, 31], one study was randomized, parallel [26]. The length of randomized studies ranged from 4 to 6 months (Table 4).

**Table 4:** Studies comparing the effect of online hemodiafiltration (HDF) and bicarbonate hemodialysis (HD) on time of recovery after dialysis in end-stage renal disease patients

	Type of study	Number of patients	Intervention	Duration	Measurement	Main results
Smith et al., 2017	Randomized, cross-over	100	Post-dilution HDF vs high-flux HD	4 months	TIRD	No overall difference TIRD between HDF and HD (median values of 47.5 [IQR, 0–240] and 30 [IQR, 0–210] minutes, respectively; *P* = 0.9
Ethier et al., 2021	Randomized, cross-over	145	HDF vs HD	6 months	TIRD	Overall, there was no difference in TIRD with the switch to HDF. HDF was associated with reduced TIRD in patients with TIRD > 60 min at baseline Median difference in TIRD (minutes) = − 120 [IQR:− 630; 0] in HDF group (*n* = 44) vs 0 [− 180; 90] in the HD group (*n* = 9); *P* < 0 .001;
Pecoits-Filho et al., 2021	Randomized, parallel	195	Post-dilution HDF vs high-flux HD	6 months	TIRD	Change from baseline to 6 months: HD = 30 [0–120] to 0 [0–60] vs HDF = 30 [0–60] to 0 [0–60]#; *P* > 0.05

*TIRD* time of recovery after dialysis

^*^TIRD was assessed on scale ranging from 0 to 5

^**^Estimated ORs for associations of HD treatment variables with patient-reported recovery time

^#^Median [IQR]

### Quality assessment

The overall score of randomized, parallel studies (n. 5) comparing the effect of HD and HDF in terms of quality of life, fatigue, and TIRD ranged from 7 to 12 (Supplementary Material Table 1S). All studies clearly stated the research question (item 1), clearly defined and used valid and reliable exposure (item 9), clearly stated if other interventions were avoided or were similar in the groups (item 10), and outcomes were assessed using valid and reliable measures, implemented consistently across all study participants (item 11). In all studies, the groups were similar at baseline on important characteristics that could affect outcomes (item 6), outcomes were reported or subgroups analyzed prespecified (item 13) and all randomized participants were analyzed in the group to which they were originally assigned (item 14). In three studies, the overall drop-out rate from the study at the endpoint was 20% or lower of the number allocated to treatment (item 7) and in four studies the differential drop-out rate (between treatment groups) at the endpoint was 15 percentage points or lower (item 8). Three studies justified the sample size (item 12). Three studies reported an adequate method of randomization (item 2) and in three studies the treatment allocation was concealed? None of the studies reported whether study participants and providers were blinded to treatment group assignment (item 4) or investigators were blinded to the exposure of participants (item 5).

The overall score of randomized, cross-over studies comparing the effect of online hemodiafiltration (HDF) and bicarbonate hemodialysis (HD) in terms of quality of life, fatigue, and TIRD was “low risk of bias” (Supplementary Material Table 2S). All studies clearly stated the research question 1. appropriate cross-over design; 2. the randomized order of receiving treatment; 3. carry-over effects; 4. unbiased data; 7. incomplete outcome data; 8. selective outcome reporting; 9. other biases. Three studies clearly stated research question 5, allocation concealment and none the research question 6, blinding.

### Efficacy of interventions on QOL

#### Comparison of HD and online HDF in terms of quality of life assessed through the PCS and the MCS of SF36 or SF12: randomized studies

**Fig. 2:**
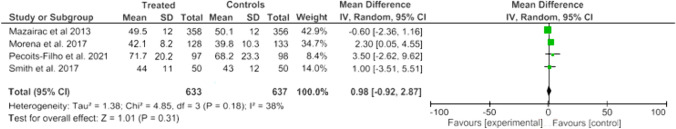
Forest plot of studies comparing HD and HDF about MCS score

**Fig. 3:**
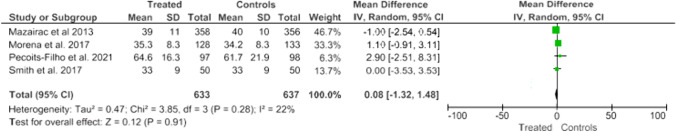
Forest plot of studies comparing HD and HDF about PCS score

Out of 5 randomized studies comparing HD and online HDF in terms of quality of life assessed through the PCS and the MCS, 4 were included in the meta-analysis [23–26]. One study was excluded because PCS and MCS were not expressed as mean (SD) difference [22]. As shown in Fig. 2, compared to HD, online HDF had a pooled MD of the MCS score of 0.98 (95% CI − 0.92, 2.87; *P* = 0.31). No significant heterogeneity was observed (Chi^2^ = 4.85; *I*^2^ = 38%; *P* = 0.18). Figure 3 shows that compared to HD, online HDF had a pooled MD of the PCS score of 0.08 (95% CI − 1.32, 1.48; *P* = 0.91). No significant heterogeneity was observed (Chi^2^ = 3.85; *I*^2^ = 22%; *P* = 0.28). The study of Kantartzi et al., not included in the meta-analysis, did not show any significant difference of the median SF36 scores between HD and HDF [22].

#### Comparison of HD and online HDF in terms of quality of life assessed through the Kidney Disease Questionnaire or KDQOL-SF: randomized studies

In the study of Ward et al. [27], patients’ assessment of physical symptoms showed a significant improvement during the study (*P* < 0.001). However, such an increase did not depend on the mode of therapy (*P* = 0.230) and none of the other dimensions of the Kidney Disease Questionnaire showed a change over the course of the study. In the study of Karkar et al. [29], 72 patients, on regular low-flux HD for 51 ± 26 month mean age of 54 ± 12 years, were randomized to HF (group 1, *n* = 36) and to HDF (group 2, *n* = 36) and followed up for 24 months. Assessment was based on the patient’s satisfaction level using modified questionnaires of the validated Kidney Disease Quality of Life-Short Form (KDQOL-SF) version 1.3. As result, the HDF group achieved a higher satisfaction level than the HF group (*P* < 0.0001) with less cramps (3 ± 5 vs. 55 ± 8), itching (9 ± 10 vs. 48 ± 10), joint pain and stiffness (24 ± 10 vs. 83 ± 8), and improvement in general mood (94 ± 9 vs. 28 ± 16), sexual performance (57 ± 10 vs. 5 ± 5), and social activity (82 ± 9 vs. 15 ± 8.). In the study of Aichi et al. [30], including 47 patients, the switch from bicarbonate hemodialysis to HDF did not lead to a significant improvement of quality of life. The study of Schiffl et al. [16], including 76 patients, hemodiafiltration was associated with a lower number of hypotensive episodes and partial improvement of quality of life.

### Efficacy of interventions on fatigue

The study of Karkar et al., including 72 patients, 24 months of treatment with high-efficiency post-dilution HDF, compared with the high-flux-treated group, showed a better effect on general fatigue (57 ± 12 with HD and 6 ± 6 with HDF; < 0.0001) and post-dialysis fatigue (61 ± 18 with HD and 10 ± 9 with HDF; < 0.0001) [29]. Assessment was based on patient’s satisfaction level using a modified questionnaire survey of the Kidney Disease Quality of Life-Short Form with a scale ranging from 0 to 100, where 0 reflects the poor effect and 100 reflects the maximum positive effect [24]. Conversely, Ward et al. have shown that fatigue significantly improved during the study comparing HD with HDF, but the increase did not depend on the therapy mode (*P* = 0.083) [27]. Similarly, in the study of Kantartzi et al. the energy/fatigue component of the SF36 did not differ significantly between patients receiving HD and HDF [22].

### Efficacy of interventions on TIRD

The study of Smith et al. recently demonstrated that there was no overall difference in TIRD between HD and HDF treatments (medians for HDF vs HD of 47.5 [IQR, 0–240] vs 30 [IQR, 0–210] minutes, respectively; *P* = 0.9) [25]. Accordingly, the longitudinal study of Pecoits-Filho et al. has demonstrated that the difference in the change in TIRD from baseline showed no significant distinctions between HD and HDF, although HDF patients reported a 38.1 min (95% CI 78.5–2.3) shorter TIRD at 3 months and 33.7 min (95% CI 79.8–12.4) shorter TIRD at 6 months [26]. Conversely, in the study of Ethier et al., the switch from HD to HDF was associated with substantially reduced recovery time in patients with recovery time > 60 min at baseline [31].

## Discussion

The present systematic review and meta-analysis showthat bicarbonate HD and online HDF are comparable in terms of quality of life in end-stage renal disease patients on maintenance hemodialysis. These results are partially in disagreement with a previous systematic review published in 2018 [32] comparing HDF versus HD that found a reduction in the PCS score that was only of 0.81 in the range of 0 to 100, and a reduction of MCS that was only 1.24 points in a range from 0 to 100. With respect to that review, in the meta-analysis comparing MCS and PCS of patients receiving HD or HDF, we excluded one small cross-over study and included the large randomized, parallel study of Pecoits-Filho et al. published in 2021 [26].

In addition, the present systematic review included studies that compared the QoL assessed through the Kidney Disease Questionnaire or KDQOL-SF and shows that HDF did not improve QoL when compared with HD, in most studies.

It is difficult to explain why HDF does not confer any benefit in terms of quality of life. It is well known, in fact, that online HDF removes an enhanced number of solutes and reduces subclinical chronic inflammation with respect to bicarbonate hemodialysis [33–35]. It has been suggested that QoL questionnaires are not sufficiently specific or sensitive tools to explore the effects of HD or HDF on patients’ perception and that simpler and more specific questions (time of recovery after dialysis and patient-reported outcome measures) have more predictive value for ESRD patients’ outcomes [7]. It is also possible that in some patients, included in the studies herein assessed, the optimal convection volume was not achieved.

The present systematic review also shows that in two out of three studies, HDF was not significantly more effective than HD in improving fatigue. This is an intriguing result, considering that HDF is commonly believed to significantly reduce the symptoms of uremic patients. Indeed, fatigue in hemodialysis patients is a multifactorial event that develops or worsens after the hemodialytic session and may persist between the dialytic sessions [36]. Its prevalence ranges from 20 to 86%, likely due to variations in methods of ascertainment and participant characteristics. Although many variables have been found to be associated with fatigue such as depression, sedentary behavior, functional disability, ultrafiltration volume, intradialytic hypotension, and branched-chain amino acid depletion, the causes of fatigue in hemodialysis patients are not clearly understood [36].

The present systematic review also demonstrates that the length of the recovery time is similar in patients receiving HDF and HD. This is the first time that such a result has been reported. Time of recovery after dialysis has been shown to be significantly associated with quality of life [16, 37], higher number of comorbidities [38], higher ultrafiltration rate [39], and depression [40].

The lack of effect of HDF in improving QoL, fatigue and TIRD observed in the present systematic review is difficult to explain considering that HDF is associated with a better removal of both small and middle-sized molecules and that, the existing evidence, suggests that it offers a 25% reduction in cardiovascular death compared with diffusive-based therapies [41]. Certainly, the present review has some limitations. First, the number of studies included in the meta-analysis as well as in the systemic review is limited. Second, the sample size of many of the included trials was small. Third, the length of the studies was extremely various and, in most trials, too short. Fourth, many studies dating back to the early 2000s, when HDF was in nascent stages, were included. Finally and most importantly, the substitution volume in patients receiving HDF ranged from 15 to 40 L/session and the dialysate flow ranged from 415 to 700 ml/min or it was not reported in many studies (Table 5). This is a critical point considering that other outcomes, such as all-cause mortality and cardiovascular mortality, have been shown to be influenced significantly by the delivered convective volume [42] and that thereafter a minimum convection volume of 23 L/1.73 m^2^ BSA per session for HDF treatments is recommended [43].

**Table 5:** Studies comparing the effect of HD and HDF: dialysate flow and substitution volume. Session intended of 4 h

		Dialysate flow (ml/min)	Substitution volume (L/session)
Kantartzi et al., 2013^5^	HD HDF	500–700	15–20
Mazairac et al., 2013^6^	HD HDF	Not reported	24
Morena et al., 2017^7^	HD HDF	510 540	19–22.5
Smith et al., 2017^8^	HD HDF	Not reported	20.6
Pecoits-Filho et al., 2021^9^	HD HDF	Not reported	22
Ward et al., 2000	HD HDF	500 415–435	15.6–20.5
Schiffl et al., 2007	HD HDF	500	20
Karkar et al., 2015	HD HDF	Not reported	19.3
Aichi et al., 2022	HD HDF	500	40
Ethier et al., 2021	HD HDF	Not reported	26–28.9

It has also to be said that QoL, fatigue and recovery time are complex and multidimensional entities, influenced by numerous demographic, clinical and laboratory variables and it remains unclear if they can be modified by a single therapeutic modality or, rather, by a multidisciplinary approach [44–47].

In conclusion, the evidence in the current literature seems to suggest that HDF, when compared to bicarbonate HD, doesn’t significantly improve the QoL and doesn’t significantly reduce fatigue and time of recovery after dialysis in end-stage renal disease patients on maintenance hemodialysis. However, the limitations of the present systematic review and meta-analysis suggest caution in the interpretation of the results. Fortunately, randomized, controlled studies on online HDF, such as CONVINCE and H4RT [48, 49], currently underway will hopefully define the role of HDF in improving QoL, fatigue and recovery time in patients on chronic hemodialysis. Nevertheless, it is possible that, in the next future, other dialytic modalities such as intensive hemodialysis or hemodialysis with medium cut-off membranes will be tested through adequate randomized trials in terms of QoL and fatigue/time of recovery.

## Supplementary Information

Below is the link to the electronic supplementary material.

Supplementary file1 (DOCX 15 KB)
