# Supplementary material for: Effect of online hemodiafiltration on quality of life, fatigue and recovery time: a systematic review and meta-analysis
Source: J Artif Organs. 2024 Jul 27;28(1):15–24. doi: 10.1007/s10047-024-01459-7 (PMC11832692; doi:10.1007/s10047-024-01459-7)
Supplement: Supplementary file 1 — Supplementary file1 (DOCX 15 KB) [file 10047_2024_1459_MOESM1_ESM.docx]

**SUPPLEMENTARY MATERIALS**

| Authors | 1 | 2 | 3 | 4 | 5 | 6 | 7 | 8 | 9 | 10 | 11 | 12 | 13 | 14 | Overall score |
| --- | --- | --- | --- | --- | --- | --- | --- | --- | --- | --- | --- | --- | --- | --- | --- |
| Ward et al, 2000 | Y | NR | NR | N | NR | Y | N | Y | Y | Y | Y | NR | Y | Y | 8 |
| Mazairac et al, 2013^6^ | Y | Y | Y | N | NR | Y | Y | Y | Y | Y | Y | Y | Y | Y | 12 |
| Morena et al, 2017^7^ | Y | Y | Y | N | NR | Y | Y | Y | Y | Y | Y | Y | Y | Y | 12 |
| Karkar et al, 2015 | Y | NR | NR | NR | NR | Y | NR | NR | Y | Y | Y | NR | Y | Y | 7 |
| Pecoits-Filho et al, 2021^9^ | Y | Y | Y | N | NR | Y | Y | Y | Y | Y | Y | Y | Y | Y | 12 |

**Table 1S**. Quality analysis of studies comparing HDF and HD in terms of QOL, fatigue, and TIRD: randomized, parallel studies.

Legend. 1. Was the study described as randomized, a randomized trial, a randomized clinical trial, or an RCT? 2. Was the method of randomization adequate (i.e., use of randomly generated assignment)? 3. Was the treatment allocation concealed (so that assignments could not be predicted)? 4. Were study participants and providers blinded to treatment group assignment? 5. Were the people assessing the outcomes blinded to the participants' group assignments? 6. Were the groups similar at baseline on important characteristics that could affect outcomes (e.g., demographics, risk factors, co-morbid conditions)? 7. Was the overall drop-out rate from the study at endpoint 20% or lower of the number allocated to treatment? 8. Was the differential drop-out rate (between treatment groups) at endpoint 15 percentage points or lower? 9. Was there high adherence to the intervention protocols for each treatment group? 10. Were other interventions avoided or similar in the groups (e.g., similar background treatments)? 11. Were outcomes assessed using valid and reliable measures, implemented consistently across all study participants? 12. Did the authors report that the sample size was sufficiently large to be able to detect a difference in the main outcome between groups with at least 80% power? 13. Were outcomes reported or subgroups analyzed prespecified (i.e., identified before analyses were conducted)? 14. Were all randomized participants analyzed in the group to which they were originally assigned, i.e., did they use an intention-to-treat analysis?

| Authors | 1 | 2 | 3 | 4 | 5 | 6 | 7 | 8 | 9 | Overall |
| --- | --- | --- | --- | --- | --- | --- | --- | --- | --- | --- |
| Kantartzi et al, 2013^5^ | Low | Low | Low | Low | Unclear | High | Low | Low | Low | Low |
| Schiffl et al, 2007 | Low | Low | Low | Low | Low | High | Low | Low | Low | Low |
| Smith et al, 2017^8^ | Low | Low | Low | Low | Low | High | Low | Low | Low | Low |
| Aichi, 2022 | Low | Low | Low | Low | Low | High | Low | Low | Low | Low |

**Table 2S**. Quality analysis of studies comparing HDF and HD in terms of QOL, fatigue, and TIRD: randomized, cross-over studies.

Legend. 1. appropriate cross-over design; 2. the randomized order of receiving treatment; 3. carry-over effects; 4. unbiased data; 5. allocation concealment; 6. blinding; 7. incomplete outcome data; 8. selective outcome reporting; 9. other biases
